# Bisphenol A Alters **β**-hCG and MIF Release by Human Placenta: An *In Vitro* Study to Understand the Role of Endometrial Cells

**DOI:** 10.1155/2014/635364

**Published:** 2014-03-11

**Authors:** C. Mannelli, F. Ietta, C. Carotenuto, R. Romagnoli, A. Z. Szostek, T. Wasniewski, D. J. Skarzynski, Luana Paulesu

**Affiliations:** ^1^Department of Life Sciences, University of Siena, Via A. Moro 2, 53100 Siena, Italy; ^2^Department of Reproductive Immunology and Pathology, Institute of Animal Reproduction and Food Research, Polish Academy of Sciences, Tuwima 10 Street, 10-747 Olsztyn, Poland; ^3^Department of Gynecology and Obstetrics, Faculty of Medical Sciences, University of Warmia and Masuria, Zolnierska 14 C Street, 10-561 Olsztyn, Poland

## Abstract

A proper fetomaternal immune-endocrine cross-talk in pregnancy is fundamental for reproductive success. This might be unbalanced by exposure to environmental chemicals, such as bisphenol A (BPA). As fetoplacental contamination with BPA originates from the maternal compartment, this study investigated the role of the endometrium in BPA effects on the placenta. To this end, *in vitro* decidualized stromal cells were exposed to BPA 1 nM, and their conditioned medium (diluted 1 : 2) was used on chorionic villous explants from human placenta. Parallel cultures of placental explants were directly exposed to 0.5 nM BPA while, control cultures were exposed to the vehicle (EtOH 0.1%). After 24–48 h, culture medium from BPA-treated and control cultures was assayed for concentration of hormone human Chorionic Gonadotropin (**β**-hCG) and cytokine Macrophage Migration Inhibitory Factor (MIF). The results showed that direct exposure to BPA stimulated the release of both MIF and **β**-hCG. These effects were abolished/diminished in placental cultures exposed to endometrial cell-conditioned medium. GM-MS analysis revealed that endometrial cells retain BPA, thus reducing the availability of this chemical for the placenta. The data obtained highlight the importance of *in vitro* models including the maternal component in reproducing the effects of environmental chemicals on human fetus/placenta.

## 1. Introduction

During pregnancy, two genetically different organisms, the mother and the fetus, establish a molecular cross-talk [1–3]. In this cross-talk, a huge number of molecules are secreted by both organisms and act on both sides [4]. The concerted action of endocrine, paracrine, and autocrine molecules makes the maternal decidua an immunologically privileged site, in which the semiallogenic embryo/fetus is allowed to grow and develop [5, 6]. A correct and timely signalling between the mother and the fetus/placenta is of paramount importance for embryo implantation and further development [7]. Hormones, such as estradiol-17*β* (E2) and progesterone (P4), play key roles in the preparation of the maternal uterine decidua for the implanting blastocyst [8, 9]. Similarly, these hormones regulate the development of the embryo/fetus and the correct process of placentation [10–12].

Among the plethora of molecules that are secreted at the fetomaternal interface, the Macrophage Migration Inhibitory Factor (MIF) is a proinflammatory cytokine which has been shown to play a regulatory role in human pregnancy. MIF is expressed by the human placenta throughout gestation and mainly in the early stages of pregnancy [13, 14]. Any variation of MIF, towards either an increase or a decrease in the maternal serum, has been associated with complications in human pregnancy, including miscarriage, preterm labour, and preeclampsia [15–18]. These data reveal the importance of MIF in human pregnancy.

The beta subunit of human Chorionic Gonadotropin (*β*-hCG) is specifically secreted by the trophoblast, starting from the very first stages of gestation [19–21]. It plays a key role in implantation and in the maintenance of pregnancy [19, 21].

Acting together, immune and hormonal stimuli lead the maternal tissues to the formation of the decidua at the end of each cycle. If fertilization has occurred, the blastocyst reaches the uterine epithelium and implantation starts. The trophoblast cells which form the epithelial covering of the blastocyst first contact the uterine epithelium and then dislodge the epithelial cells and invade the maternal endometrium [22]. *β*-hCG is the signal of human pregnancy capable of preventing the corpus luteum from degenerating, thus allowing blastocyst implantation and placenta development. Acting on the human endometrium, MIF is able to reduce the cytotoxicity of decidual NK cells, thus suggesting an important role in the maternal immunotolerance versus the semiallogenic fetus [23]. Any unbalance in *β*-hCG and MIF secretion could have serious consequences for pregnancy and fetal health.

Bisphenol A (BPA) is the prototype of environmental chemicals, with a well-recognized hormone-like activity and wide diffusion in several items, including polycarbonate plastics and cans used for food and beverages [24]. As any other human being, women in reproductive age and mothers during pregnancy can be daily exposed to BPA. Contamination by this chemical can occur through dermal absorption by using personal care products and detergents, breathing contaminated air, and drinking or ingesting contaminated water or food [25, 26]. BPA can bind the receptors of endogenous steroid hormones and thus is able to interact with the hormone target organs [27–32]. Due to its high lipophilic property, BPA can be transferred all through the placenta and reach the fetus [33]. Animal studies showed that pre- and perinatal exposure to this chemical causes problems to the newborn such as low birth weight and/or predisposition to adult degenerative diseases, that is, cardiovascular diseases, diabetes, obesity, and allergies [34–36]. BPA acts also in human placenta at concentrations as low as nM [33].

As fetoplacental contamination with BPA originates from the maternal compartment, this study aimed to investigate the effect of maternal contamination with BPA on the human placenta. In the attempt to resemble the complex molecular interactions between the mother and the fetus in early pregnancy, a cell-free strategy with conditioned medium from endometrial stromal cells was used to expose placental cultures from first trimester of pregnancy. MIF and *β*-hCG were assayed as key molecules of the endocrine-paracrine secretions for placenta establishment and development.

## 2. Materials and Methods

This study was conducted within a collaborative project between the Laboratory of the Department of Reproductive Immunology and Pathology, Institute of Animal Reproduction and Food Research, Polish Academy of Sciences, Olsztyn, Poland, and the Laboratory of the Department of Life Sciences, University of Siena, Italy. If not otherwise specified, the procedures were carried out in both laboratories.

### 2.1. Isolation and Culture of Endometrial Stromal Cells

Biopsies (of about 0.5 cm^3^ each) from healthy human endometrium (in total *n* = 3 specimens from different donors, all from early proliferative phase of cycle) were obtained from the Prefectural Hospital of Olsztyn (Poland) (*n* = 2 specimens) and from the Hospital of Campostaggia (Siena, Italy) (*n* = 1 specimen) after written informed consent of the patients and with the approval of the local Ethics Committee (490/12/BIOET, for the Hospital of Olsztyn, and VITRO-RIP 2013, for the Hospital of Campostaggia), in accordance with the Helsinki Declaration guidelines. Dating of the endometrial tissue was performed according to the date of the last menstrual period and to the standard histological dating performed in the hospital [37]. Stromal cells were isolated as described by Hombach-Klonisch et al. [38] with some modifications. Briefly, the tissue was cut into 1 mm^3^ pieces and incubated in DMEM-F12 medium, without phenol red (Sigma-Aldrich, St. Louis, MO, USA), supplemented with 1% antibiotic/antimycotic (Sigma-Aldrich), 0.1% (w/v) albumin from Bovine Serum (BSA) (Roche Diagnostics GmbH Mannheim, Germany), 2.4 IU/mL dispase (Gibco, Grand Island, NY, USA), 0.05% (w/v) collagenase (Sigma-Aldrich), and 0.005% DNase I (Sigma-Aldrich) for 20 minutes, at 37°C, with gentle shaking. After the enzymatic digestion, stromal cells were separated from epithelial cells with nylon strainers of 70 *μ*m (Becton Dickinson, Falcon, Franklin Lakes, NJ, USA) and finally 40 *μ*m (BD Falcon) of pore size. Cells were then centrifugated at 100 ×g for 10 minutes at 4°C and the erythrocytes present in the cell pellet were lysed by addition of the red blood cell lysis buffer (Sigma-Aldrich). The cells were finally resuspended in phenol red-free DMEM-F12 (Lonza BioWhittaker, Verviers, Belgium) supplemented with 10% Fetal Bovine Serum (FBS) (Sigma-Aldrich) and 1% antibiotic/antimycotic (Sigma-Aldrich) and seeded in 6-well plates (BD Falcon). Medium containing unattached cells was removed after 2-3 h, in order to further purify the culture. After reaching 70–80% confluence, cells were then released from the plates by trypsin/EDTA (Lonza BioWhittaker) and reseeded, at a concentration of 1 × 10^6^ cells/mL, in 25 cm^2^ flasks (BD Falcon).

### 2.2. Characterization and Decidualization of Endometrial Stromal Cells

Endometrial stromal cells were cultured in humidified atmosphere of 5% CO_2_ in air, at 37°C in complete DMEM-F12 medium, without phenol red, supplemented with 10% FBS and 1% antibiotic/antimycotic, until 70–80% confluence. The purity of the stromal cells from each specimen was confirmed by flow cytometry and immunocytochemistry as described below. The cells were then decidualized by priming with steroid hormones, resembling the conditions of the cycling endometrium [39, 40]. In particular, cells in 25 cm^2^ flasks at 70–80% confluence (about 1.3 × 10^6^ cells/flask) were cultured without hormones for the first three days [38]; then, in order to mimic the proliferative phase of the cycle, the medium was supplemented with E2 (10^-8 ^M) (Sigma-Aldrich) and cultures incubated for a further 3 days. Finally, the cells were exposed to E2 + P4 (10^−8 ^M/10^−6 ^M, resp.) (Sigma-Aldrich) up to 12 days to mimic the secretory phase [40]. Cell decidualization was confirmed by assaying the secretion of the Insulin-Like Growth Factor Binding Protein-1 (IGFBP-1) by an Enzyme-Linked Immunosorbent Assays (ELISA). The cells were harvested in TriReagent (Sigma-Aldrich) for normalization of IGFBP-1 concentration with total DNA content.

### 2.3. Isolation and Culture of Placental Villous Explants

First trimester human placenta samples (*n* = 3 specimens) were obtained after elective termination of pregnancy at weeks 8–10 of gestation from the Hospital of Campostaggia (Siena, Italy). Collection of tissues was performed after written informed consent of the patients and with the approval of the local Ethics Committee (VITRO-RIP 2013), in accordance with the Helsinki Declaration guidelines. The tissues were brought to the laboratory and processed within a maximum of 2 h after collection. The samples were rinsed in cold serum-free DMEM-F12 without phenol red (Lonza BioWhittaker) and supplemented with 1% of penicillin/streptomycin (Sigma-Aldrich) and with 1% of L-glutamine (Sigma-Aldrich), to remove excessive blood. Villous explants were dissected as previously described [13]. Briefly, small fragments of villous tips (15–20 mg wet weight) were isolated and placed on Millicell-CM culture dish inserts (Millipore Corp., Bedford, MA) previously coated with 180 *μ*L of undiluted Matrigel (Collaborative Biomedical Products, Bedford, MA) and then transferred to 24-well culture plates. The explants were maintained overnight in 250 *μ*L of serum-free DMEM-F12 in a humidified 20% O_2_ and 5% CO_2_ atmosphere at 37°C to allow the attachment to the Matrigel. The next day, the culture medium in the outer chamber of the well was replaced with fresh medium containing the treatments and 250 *μ*L of medium containing the treatments was added to the transwells. This part of the study was conducted in the Laboratory of the Department of Life Sciences, University of Siena (Italy).

### 2.4. Experimental Procedures

#### 2.4.1. Conditioned Medium from* In Vitro* Decidualized Stromal Cells

*In vitro* decidualized stromal cells from single donors were seeded in 25 cm^2^ flasks (BD Falcon). The cells were used for the experiments at the second passage of culture. When a confluence of 80–90% was reached, cells were maintained in a medium devoid of hormonal stimuli (phenol red-free DMEM-F12 plus 1% antibiotic/antimycotic plus 0.1% Bovine Serum Albumin, BSA) for 24 h. Then, cultures (about 1.3 × 10^6^ cells per flask) were exposed to 1 nM BPA (Sigma-Aldrich) and, further, incubated for 24 h. Control cultures were exposed to the vehicle (EtOH 0.1%), as BPA was dissolved in EtOH as well. BPA treatments and control cultures were carried out in duplicates, in separated 25 cm^2^ flasks. At the end of the experiment, the culture medium of both, BPA-treated and control cultures, was separately collected in pyrogen-free sterile tubes and immediately centrifuged at 13000 ×g at 4°C for 10 minutes. The supernatant was subdivided in aliquots and kept at −80°C until use for treatment on placental explants (conditioned medium from stromal cells). Cells were harvested in ice cold RIPA lysis buffer (Tris base 40 mM, NaCl 150 mM, EDTA 500 nM, Triton X-100 1%, sodium deoxycholate 0.5%, and SDS 0.1%), containing protease inhibitor cocktail complete (Roche), for protein extraction. In total, three experiments were performed, each one using endometrial tissue from a single donor.

#### 2.4.2. Treatment of Placental Villous Explants

Villous explants were exposed to the following media:

- C: phenol red-free DMEM-F12, supplemented with 0.05% BSA;
- c-C: conditioned medium from stromal cells previously exposed to the vehicle alone; this medium was diluted 1 : 2 with fresh DMEM-F12 (supplemented with 0.1% BSA) before being used on the placenta explants;
- c-BPA: conditioned medium from stromal cells previously exposed to 1 nM BPA; this medium was diluted 1 : 2 and used as above described;
- BPA: phenol red-free DMEM-F12, supplemented with 0.05% BSA and added with 0.5 nM BPA.

In total, *n* = 3 experiments were performed, each one using placenta explants from a single donor. BPA treatments and control cultures were carried out in duplicates; samples from single explants were collected and processed separately. At 24 h of treatment, 30 *μ*L of medium was collected, centrifuged at 13000 ×g at 4°C for 10 minutes, and stored at −80°C until MIF and *β*-hCG assay. Cultures were added with an equal volume (30 *μ*L) of the respective exposure medium and incubated for a further 24 h. At the end of the experiments (48 h), the total medium was collected, centrifuged, and stored −80°C until MIF and *β*-hCG assay. Villous explants were removed from the Matrigel and immersed in ice cold RIPA lysis buffer for protein extraction.

#### 2.4.3. Measurement of Free BPA in the* In Vitro* Decidualized Stromal Cell Cultures

In order to estimate the amount of free BPA in the media, 250 *μ*L of cell suspension at a concentration of 1 × 10^6^ cells/mL of* in vitro* decidualized stromal cells was seeded in 25 cm^2^ flasks (BD Falcon). When a confluence of 80–90% was reached, cells were maintained in starvation (devoid of hormonal stimuli, supplemented with 0.1% BSA) medium for 24 h. The cells were then exposed to EtOH 0.1% (C) or to BPA (BPA 1 *μ*M; BPA 1 nM) in starvation medium and incubated for a further 24 h. In each set of samples (C, BPA 1 *μ*M, BPA 1 nM) medium was assayed for free BPA either, before (M) and after (T) exposure to the cells. The cells were washed twice with warm Phosphate Buffered Saline (PBS), added with new starvation medium, devoid of BPA, and incubated for a further 24 h. At the end of this incubation time, medium (W) and cell lysate (R) were centrifuged at 13000 ×g at 4°C for 10 minutes and stored at −80°C until BPA assay. Samples of starvation medium and RIPA buffer that did not come into contact with cells were also collected and used as blank samples. The amount of free BPA in the samples was measured by gas chromatography coupled with mass spectrometer (GC-MS).

### 2.5. Methods

#### 2.5.1. Flow Cytometry

Flow cytometric analysis was conducted using the BD FACSAria II flow cytometer one day after the isolation of a new specimen. After isolation, cells (about 4 × 10^6^ cells/flask) were plated in 75 cm^2^ flasks with complete medium (DMEM-F12, 10% FBS, and 1% antibiotic/antimycotic). The day after, cells were washed two times in warm sterile PBS and detached with Accutase 5x (cell detachment solution, Thermo Electron). An amount of at least 1 × 10^6^ cells/mL was used for the flow cytometric analysis. The cell suspension was washed twice in PBS and divided into test tubes for immunolabeling. In order to find putative impurities in the cell culture, cells were incubated in the dark for 30 minutes with fluorescein isothiocyanate- (FITC-) conjugated monoclonal antibodies against CD31(PECAM-1) (number F8402, Sigma-Aldrich), a marker of endothelial cells, and CD326 (EpCAM) (number 342203, BioLegend), a marker of epithelial cells. The control test tube was not stained.

#### 2.5.2. Immunocytochemistry

The purity of endometrial cell cultures obtained was also evaluated in each specimen, by using immunofluorescent staining for a specific marker of stromal cells (vimentin) and a specific marker of epithelial cells (cytokeratin) as described previously [41] with some modifications. 250 *μ*L of cell suspension (1 × 10^5^ cells/mL) was seeded on Thermanox Plastic Coverslips, in 4-well plate (ThermoScientific Nunc, Rochester, NY, USA) in complete DMEM-F12 medium and incubated in humidified atmosphere of 5% CO_2_ in air, at 37°C until confluence (about 5 days of culture). Cells were fixed in paraformaldehyde. After that, nonspecific binding was avoided by incubation with rabbit serum (Sigma-Aldrich). Then, slides were incubated with primary antibody against vimentin (Sigma- Aldrich) or against cytokeratin (Sigma-Aldrich). A Cy3 labeled antibody (Sigma-Aldrich) was used as secondary antibody. Coverslips were mounted in Vectashield with DAPI (Vector Laboratories, Inc., Burlingame, CA). Human endometrial stromal cells were observed under inverted confocal microscope (Nikon Eclipse Ti-E).

#### 2.5.3. Tissue and Cell Lysates

Total lysates of placental explants or endometrial stromal cell cultures were harvested in RIPA lysis buffer and sonicated on ice. Total lysates were centrifuged at 12,000 ×g for 15 min at 4°C and stored at −80°C until use. The lysates from placental explants were assessed for total protein concentration by Bradford Protein Assay (BioRad), while the lysates from endometrial stromal cell were assessed for BPA content by Gas Chromatography coupled with Mass Spectrometry (GC-MS).

#### 2.5.4. ELISA Assays

*IGFBP-1*. IGFBP-1 analysis was performed in the culture medium of endometrial stromal cells as a marker of decidualization by using a commercial ELISA assay (R&D Systems). This was done following the manufacturer's instructions. Briefly, the plates were coated with the capture antibody overnight and, the day after, nonspecific binding was avoided by incubation with blocking solution (10 mM PBS, pH 7.4, 5% (vol/vol) Tween 20) at room temperature (RT). The standard and samples were added in duplicates. After washing, the detection antibody was added and the plates incubated at RT. Plates were supplied with streptavidin horseradish peroxidase and then with the substrate solution and finally incubated for 20 minutes at RT in the dark. The reaction was stopped by adding the stop solution (2 N, H_2_SO_4_). The optical density of each well was determined at 450 nm by a microplate reader (ThermoScientific). The concentration of IGFBP-1 was extrapolated by the software Genesis from a standard curve ranging from 12.5 to 2000 pg/mL, using human recombinant IGFBP-1 (R&D Systems) as standard. The data were normalized against the total cell DNA content, measured by NanoDrop (ThermoScientific). The IGFBP-1 concentration was expressed as pg/*μ*g of total DNA.

*MIF*. In order to detect the amount of MIF released in the medium of villous explants, a colorimetric sandwich ELISA, previously set up in our laboratory, was used [13]. 96-well plates were coated overnight at RT with anti-human MIF monoclonal antibody (2 *μ*g/mL; R&D Systems). The plates were then washed with washing solution (10 mM PBS, pH 7.4, 0.05% (vol/vol) Tween 20), blocked by the addition of 300 *μ*L of blocking solution (10 mM PBS, pH 7.4, 1% (wt/vol) BSA 5% (wt/vol) sucrose), and incubated at RT for 1.5 h. The standard and the samples were added in duplicates (100 *μ*L/well) and incubated for 2 h at RT. The plates were then washed, and 100 *μ*L of biotinylated goat anti-human MIF antibody (200 ng/mL; R&D Systems) was added to each well. Plates were incubated for 2 h at RT and then washed. Streptavidin horseradish peroxidase (Zymed, San Francisco, CA) was subsequently added to each well, and a 20 min incubation at RT followed. The plates were washed and 3,3,5,5-tetramethylbenzidine (Zymed) was added to each well; the reaction was stopped after 20 min by the addition of 2 N H_2_SO_4_. Absorbance was measured at 450 nm using a microplate reader (ThermoScientific). The MIF concentration was extrapolated from a standard curve ranging from 25 to 2500 pg/mL of human recombinant MIF (R&D Systems) as standard. The sensitivity limit of the assay was 18 pg/mL. Intra- and interassay coefficients of variation were 3.86 (0.95) and 9.14 (0.47) %, respectively. The MIF concentration was expressed as pg/mg of total protein content from its respective placental explant, measured by Bradford assay (BioRad).

**β*-hCG*. The concentration of the secreted *β*-hCG in the media of the villous explants was assessed by a commercial immunoenzymometric assay following the manufacturer's instructions (Radim SpA, Pomezia, Italy). Standard with recombinant human *β*-hCG and samples were added in duplicates to precoated 96-well plates. The enzymatic conjugate (monoclonal anti-hCG antibody horseradish peroxidase-labeled, diluted in Tris-HCl and BSA) was then added and incubated for 30 minutes at RT. After washing, the plate was incubated with the substrate solution. The reaction was blocked by the addition of blocking reagent and absorbance was measured at 450 nm using an ELISA SR 400 microplate reader (Sclavo, Siena, Italy). The *β*-hCG concentration was extrapolated from a standard curve ranging from 12.5 to 2000 mIU/mL of human recombinant *β*-hCG (Radim SpA) as standard. The limit of sensitivity was 2 mIU/mL and the linear range of detection was 0–2000 mIU/mL. The data obtained in each sample were expressed as mIU/mg of total protein content from its respective placental explant, measured by Bradford assay (BioRad).

#### 2.5.5. GC-MS

GC-MS was used for the determination of BPA in the tested samples. Quantitative analysis was done using the technique of isotope dilution mass spectrometry (IDMS). Calibration phase required the determination calibration factor (Response Factor: RF) for radiolabeled and naturally occurring standard:

(1) $$\begin{array}{c} \text{RF}=\frac{A_{\text{nat}}*C_{\text{isotop}}}{A_{\text{isotop}}*C_{\text{nat}}}, \end{array}$$

 where *A*_isotop_ is ion peak area for isotopically labeled analyte (d_16_ BPA) present in the standard sample, *A*_nat_ is ion peak area of a naturally occurring analyte (BPA) present in the sample, *C*_isotop_ is d_16_ BPA concentration in the standard sample, and *C*_nat_ is BPA concentration in the sample.

The calibration procedure was carried out using high purity water, and the blank samples were included in the calculation of the data. The values of the Response Factor (RF) were determined by using standard solutions containing known concentration of isotopically labeled BPA analogue and nine different concentrations of BPA analyte. A known amount of deuterated BPA standard was added to each analyzed sample. Then, the concentration of analyte in the sample was calculated using the designated RF values, the value of the area was obtained for the analyte in the sample, and isotopically labeled standard was added to the sample.

The quantitative analysis by GC-MS was performed in the monitoring mode (SIM) of the two ions for BPA and two ions BPA-d_16_ at different time intervals. The average blank value of the obtained results is 1.58 ± 0.13 ng/mL. This part of the study was conducted in the Laboratory of the Department of Reproductive Immunology and Pathology, Institute of Animal Reproduction and Food Research, Olsztyn, Polish Academy of Sciences, Poland.

### 2.6. Statistical Analysis

Data were analyzed on GraphPad Prism Version 5.0 (GraphPad Software, Inc., San Diego, CA). All data on *β*-hCG and MIF are shown as percentage mean ± SD versus C. The data obtained from the GC-MS analysis are shown as ng/mL ± SD, while the data from IGFBP-1 analysis are shown as pg/*μ*g total DNA ± SD. All data were statistically analyzed by the nonparametric Kruskal-Wallis test. Differences were considered significant if *P* ≤ 0.05.

## 3. Results

### 3.1. Validity of the Cultures Used

**Figure 1:**
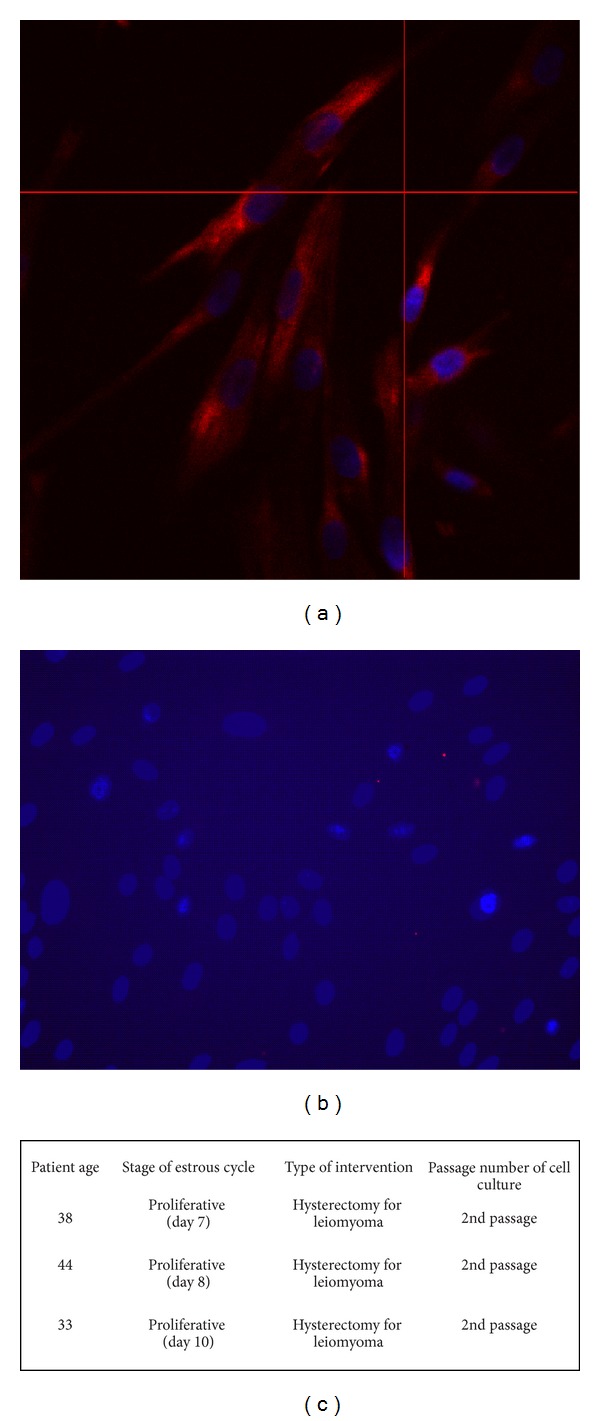
Characterization of human endometrial stromal cells by immunofluorescence. (a) Cells were stained with anti-vimentin antibody (red staining). Nuclei were stained with DAPI (blue staining). (b) Cells were negative for staining with anti-cytokeratin antibody. Only the nuclei, stained with DAPI (blue), were visible. Scale bar = 50 *μ*m. (c) Table showing the information about the origin of the endometrial specimens used in the study.

**Figure 2:**
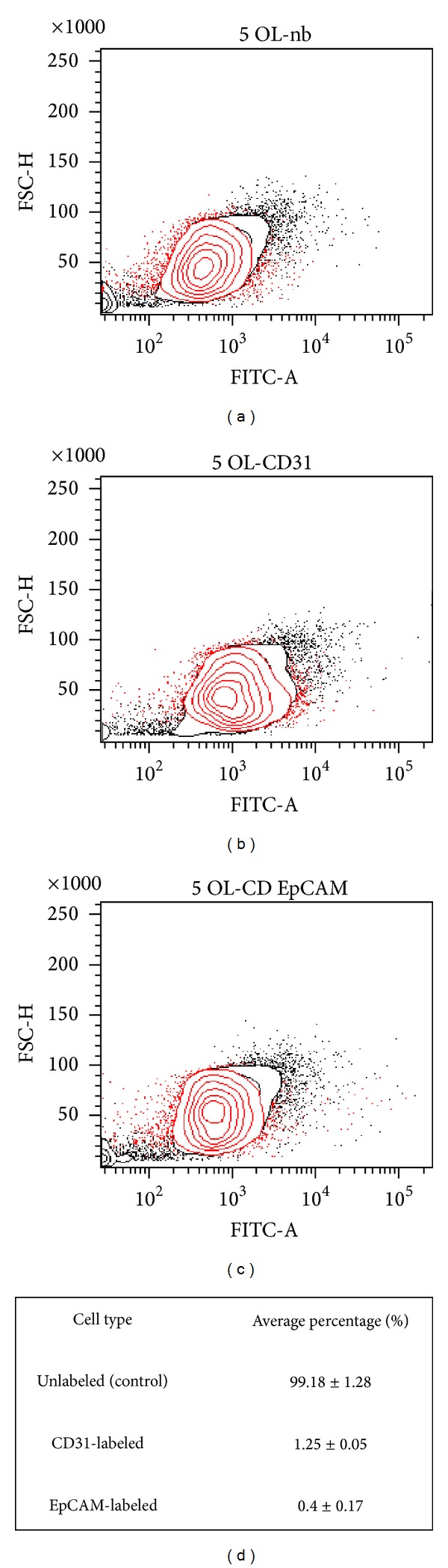
Representative flow cytometric plots of human endometrial cells, at 1st passage (day 1 after isolation). (a) Unlabeled cells. The contour plot shows the unstained population of cells. (b) Population of CD31 (EpCAM-1) stained cells, marker of endothelial cells. (c) Population of CD326 (EpCAM) stained cells, marker of epithelial cells. Data were elaborated with BD FACSAria II Software. (d) Table of the expression of the cell types expressed as average percentage ± SD of 3 endometrial specimens.

**Figure 3:**
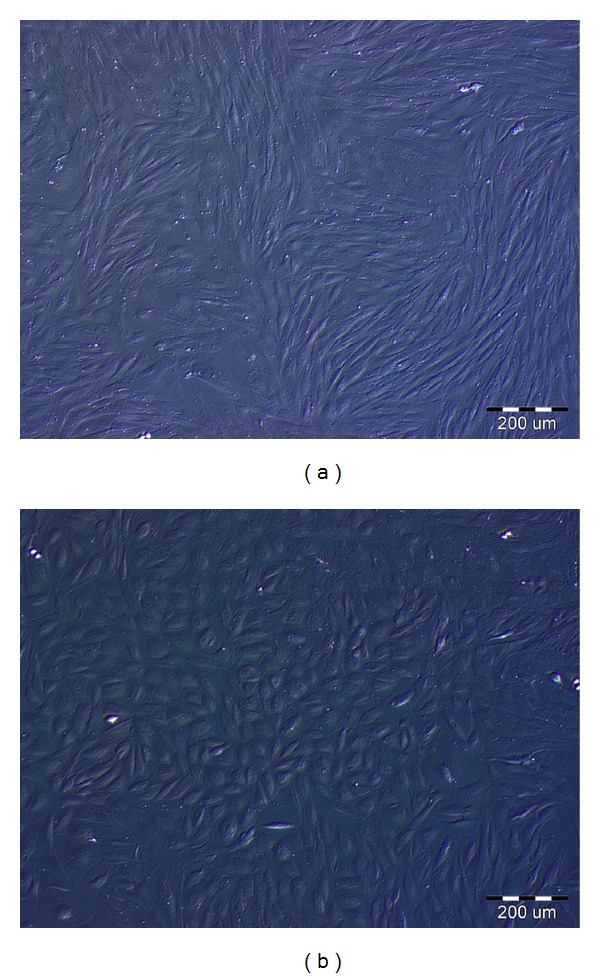
Representative pictures of human endometrial stromal cells observed under inverted microscope (Nikon). (a) Endometrial stromal cells not exposed to hormonal stimuli. The characteristic spindle-like shape of stromal cells is recognizable. (b) Endometrial stromal cells after priming with steroid hormones (E2 + P4 for 12 days). The polygonal shape, typical of decidualized cells, is recognizable. Scale bar = 200 *μ*m.

The purity of primary stromal cells from human endometrium was assessed by immunocytochemistry using antibodies against vimentin (a marker of stromal cells) and cytokeratin (a marker of epithelial cells). As shown in Figure 1, about 100% cells were immunostained with anti-vimentin (A), while no evidence of immunostaining was shown with anti-cytokeratin antibody (B). The table in Figure 1(c) summarizes the information about the three endometrial specimens used in this study. Flow cytometry was used to evaluate the percentage of contaminating endothelial (anti-CD31 antibody) and epithelial (anti-EpCAM antibody) cells (Figure 2). Analysis of all endometrial specimens (*n* = 3) revealed an average percentage of about 1.25 ± 0.05% of endothelial cells and 0.4 ± 0.17% of epithelial cells (Figure 2). The differentiation of stromal cells into a decidual phenotype was monitored throughout the culture by checking the morphology of the cells that exhibited a polygonal phenotype after 10 days of priming with E2 + P4 (10^-8 ^M/10^-6 ^M) (Figures 3(a) and 3(b)). Furthermore, decidualization of stromal cells was confirmed by assaying the release of a decidualization marker, IGFBP-1, into the culture media. This marker was selected because endometrial stromal cells start to secrete IGFBP-1, as well as prolactin, only when they differentiate into decidual cells. Indeed, IGFBP-1 is highly secreted by the endometrium during pregnancy and seems to be involved in regulating the migration of human trophoblast [42].

During the culture, endometrial stromal cells started to secrete a considerable amount of IGFBP-1 already after 4–6 days of priming with E2 + P4 (10^−8 ^M/10^−6 ^M), reaching a peak after 12 days of hormonal stimulation (with an average value of secreted IGFBP-1 of 14.27 ± 3.63 pg/*μ*g total DNA on the 12th day of culture).

As to the villous explants, their viability and functionality throughout the culture was confirmed by both secretion of *β*-hCG and the* de novo* formation of cellular outgrowths of extravillous trophoblast during the culture period as described by Genbacev et al., 1993 [43].

### 3.2. Endometrial Contribution to BPA Effects on Placental Secretion of *β*-hCG and MIF

Explants of placental villi were examined for the effect of BPA by both direct and indirect (endometrial-mediated) exposure. In this study, 0.5–1 nM BPA were selected, as representative concentrations of the amount of BPA detected in human fluids [44–46]. Furthermore, such range of concentration proved to be nontoxic for the placental tissues [33].

**Figure 4:**
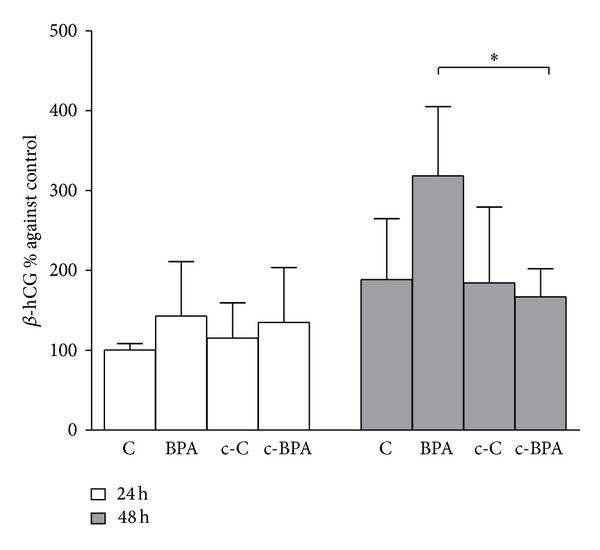
Concentration of *β*-hCG in the culture media of explants of chorionic villi exposed to EtOH 0.1% (C), 0.5 nM BPA (BPA), or conditioned medium (diluted 1 : 2) from decidualized stromal cells previously exposed to vehicle (c-C) or to 1 nM BPA (c-BPA). The time course lasted for 24 h (white bars) and 48 h (grey bars). Values were normalized against villous explant total protein content and expressed as percentage ± SD versus C at 24 h of *n* = 3 separate experiments (*P < 0.05 BPA 48 h versus c-BPA 48 h).

**Figure 5:**
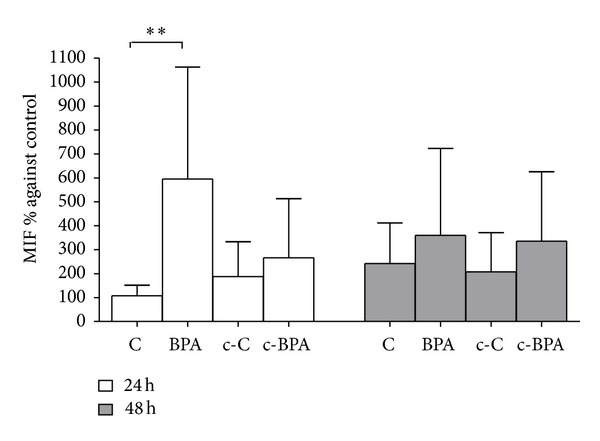
Concentration of MIF in the culture media of explants of chorionic villi exposed to EtOH 0.1% (C), 0.5 nM BPA (BPA), or conditioned medium from decidualized stromal cells previously exposed to vehicle (c-C) or to 1 nM BPA (c-BPA). The time course lasted for 24 h (white bars) and 48 h (grey bars). Values were normalized against villous explant total protein content and expressed as percentage ± SD versus C at 24 h of *n* = 3 separate experiments (**P ≤ 0.01 BPA 24 h versus C 24 h).

Figure 4 shows *β*-hCG concentration in the culture medium of placental explants treated with BPA in medium either conditioned or not conditioned by endometrial stromal cells. Data were reported as percentage mean ± SD versus the control. Control placental explants secreted increasing levels of *β*-hCG from 24 to 48 h, thus confirming the endocrine functional activity of the trophoblast during the culture time. Direct exposure to 0.5 nM BPA significantly stimulated the release of *β*-hCG at 48 h (*P* < 0.01 versus C at 24 h). This increase was also statistically significant in comparison to 48 h treatment with conditioned medium from endometrial stromal cells previously exposed to BPA (c-BPA) (*P* < 0.05 BPA 48 h versus c-BPA 48 h) (Figure 4). No significant differences were observed between the medium conditioned or not conditioned by endometrial stromal cells at 24 h treatment (Figure 4).  Figure 5 shows MIF concentration (percentage mean ± SD versus C) in the culture medium of placental explants treated with medium conditioned or not conditioned by endometrial stromal cells. Direct exposure to 0.5 nM BPA significantly triggered MIF secretion at 24 h (*P* < 0.01 versus C). The effect was lower in explants exposed to conditioned medium from decidualized stromal cells previously treated with 1 nM BPA (c-BPA).

### 3.3. BPA Levels in Decidualized Stromal Cell Cultures

**Figure 6:**
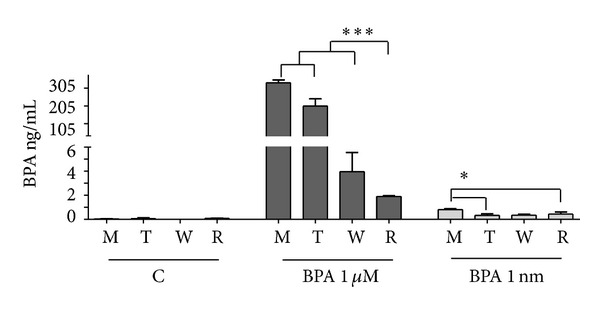
Concentration of free BPA in the culture media and cell lysates of decidualized stromal cells, detected by GC-MS. C: exposure media containing vehicle (0.1% EtOH); BPA 1 *μ*M and BPA 1 nM: exposure media containing BPA 1 *μ*M or BPA 1 nM; M: media before exposure to the cells; T: media incubated for 24 h with the cells; W: washing media incubated for 24 h with cells previously exposed to vehicle (group C) or to BPA (groups BPA); R: cell lysates in RIPA buffer, after incubation with washing medium. Values are expressed in ng/mL ± SD of free BPA of *n* = 3 separate experiments (*P < 0.05 versus M; ***P < 0.001 versus M).

In order to clarify the putative reason for the different effects obtained in placental explants between direct and indirect exposure to BPA, the amount of free BPA in stromal cells or in the culture medium was analyzed by GC-MS (Figure 6).

For this analysis, we measured free BPA in three different sets of samples: cells exposed to vehicle alone (C), to BPA 1 *μ*M, and to BPA 1 nM (see Figure 6). In each set of samples, the medium was assayed before the contact with endometrial cells (M) and after the exposure of cells for 24 h (T). The exposed cells were then washed with PBS and further incubated with medium devoid of BPA for 24 h; at the end of the incubation, the medium (W) and the cell lysates, obtained in RIPA buffer (R), were analyzed.

As shown in Figure 6, no BPA was detected in the samples containing only the vehicle (C), while the exposure media with BPA proved to have about the wanted concentration of BPA, with an average value of 334.95 ± 16 ng/mL (approximately 1.47 *μ*M) for BPA 1 *μ*M and 0.807 ± 0.06 ng/mL (approximately 3.53 nM) for BPA 1 nM (Figure 6). After exposure on cells, levels of free BPA in the culture medium decreased significantly to 204 ± 40 ng/mL, for 1 *μ*M BPA (*P* < 0.001 T versus M), and to 0.317 ± 0.08 ng/mL, for 1 nM BPA (*P* < 0.05 T versus M). Interestingly, the medium obtained from cells first washed and then incubated with medium devoid of BPA (W) contained 3.95 ± 1.59 ng/mL in the cultures previously treated with BPA 1 *μ*M (*P* < 0.001 W versus M) and 0.347 ± 0.09 ng/mL in the cultures previously incubated with BPA 1 nM. The washed cells (R) also retained a significant amount of BPA, which corresponded to 1.89 ± 0.05 ng/mL, for cultures previously incubated with BPA 1 *μ*M (*P* < 0.001 R versus M), and to 0.439 ± 0.16 ng/mL, for cultures previously incubated with BPA 1 nM (*P* < 0.05 R versus M).

## 4. Discussion

This study showed BPA effects at the fetomaternal interface. Endometrial stromal cells were selected as a model of the maternal compartment because of the hemochorial type placentation in humans. This type of placentation implies in fact that, after the dislodgement of the epithelial cell layer, the human trophoblast invades the decidual compartment taking intimate contact with the stroma and the cells that compose it. The fetal component is represented by* ex vivo *explants of human chorionic villi from first trimester gestation, a physiological model reflecting placenta establishment and development in the maternal uterus. In this study, cultures of placental explants were exposed to 0.5 nM BPA or to conditioned medium from endometrial stromal cells previously exposed to 1 nM BPA (the medium was diluted 1 : 2 vol/vol in fresh medium). Endometrial stromal cell-conditioned medium was used to mimic maternal-mediated contamination with BPA. The range of BPA concentrations here selected is consistent with epidemiological studies which show that BPA is found in human fluids in a range of 0.4–30 nM [44–46]. Our study revealed two major findings.

1. Endometrial stromal cells are able to “entrap” BPA and thus make it less available to the placental explants.
2. Endometrial stromal cells can become a source of BPA if their surrounding environment is devoid of this chemical.

Both findings are discussed in detail below.

(1) In our study, hormone *β*-hCG and cytokine MIF, two molecules that play key roles in the interactions between fetal and maternal tissues, were selected as endpoints of BPA activity in human placenta. The results showed that direct exposure to BPA stimulated the release of both markers, *β*-hCG and MIF. On the other hand, exposure to conditioned medium from endometrial stromal cells previously exposed to BPA did not result in any significant effect on placental secretion of *β*-hCG and MIF. This might suggest a protective role of endometrial cells against BPA on human placenta. To clarify the contribution of endometrial stromal cells in minimizing BPA effects on the placenta, we assessed the amount of BPA in the culture media collected from endometrial cells/culture medium. GC-MS analysis revealed that, after exposure on cells for 24 h, BPA levels in the culture medium were reduced with respect to the given dose. This might indicate that the diminished response of villous explants on exposure to endometrial cell-conditioned medium was due to a lower availability of BPA. Moreover, the data indicate that BPA is absorbed or metabolized by endometrial stromal cells. Such hypothesis is supported by animal studies showing that the rat uterine endometrium is able to sequester and metabolize BPA, thus protecting the fetus [47]. Metabolic transformation of BPA occurs in the liver by glutathione transferases and cytochrome P450 enzymes [48, 49]. These enzymes are present in the human endometrium and thus could contribute to BPA metabolism [50–52].

(2) Endometrial stromal cells after exposure to BPA can become a source of this chemical. When the cells, first exposed to BPA, were cultured in a BPA-free environment, BPA was detected in the culture medium. In agreement with the lipophilic nature of BPA [53], the findings indicate diffusion towards the inside or towards the outside of the cell, following concentration gradient. On these bases, it could be hypothesized that human endometrium accumulates BPA in everyday life and may release BPA to the fetal compartment during pregnancy. In view of the data here collected, it is important to underline that BPA is transferred through the placenta [33]. Transport through fetal membranes has been also demonstrated by the presence of BPA in fetal tissues and umbilical cord blood [48].

The cell-free strategy here used does not exclude that molecules potentially induced by BPA and present in the culture medium of stromal endometrial cells could also play a protective role in the placenta. Further studies are required in order to elucidate this issue.

Using the same model of placental explants, we previously showed that 1 nM para-nonylphenol (*p*-NP), an estrogen-like chemical, sharing many of its properties with BPA, had similar effects to the ones observed here, resulting in the induction of *β*-hCG and upregulation of a panel of cytokines, statistically significant for GM-CSF and IL-10 [54, 55]. The previous and the present data reveal that estrogen-like chemicals, that is, *p*-NP and BPA, unbalance endocrine-paracrine secretion at the fetomaternal interface. BPA concentrations, namely, 1 nM for endometrial stromal cells and 0.5 nM for the placenta, are within the average concentrations found in human tissues and fluids. With regard to pregnancy, levels detected in maternal and fetal fluids ranged from 1 to 36 nM [18, 46].

Furthermore, to the best of our knowledge, this study showed for the first time the role of the fetal-maternal interaction in overcoming the effects of an exogenous substance such as BPA on human placenta. Indeed, we showed that direct exposure to BPA triggered placental secretion of MIF and *β*-hCG. These effects were abolished in the presence of conditioned medium from endometrial cells previously treated with BPA. *β*-hCG promotes MIF secretion in the human endometrium and exerts positive effects, that is, by stimulating angiogenic molecules such as VEGF [56, 57]. It can be thus expected that *β*-hCG modulates MIF secretion in the human placenta. This could explain the similar trend observed for these two markers. Under physiological conditions, the secretion of *β*-hCG is stimulated by estrogens, while it is inhibited by progesterone [58]. *β*-hCG promotes differentiation and invasion of the trophoblast and has immunomodulatory properties [21]. The excessive release of *β*-hCG upon exposure to BPA raises concern on the effects that this chemical could exert at the fetomaternal interface. Indeed, increased levels of *β*-hCG have been reported in gestational pathologies, such as preeclampsia [59, 60]. Similarly, concentrations of MIF in maternal serum during pregnancy are critical for the reproductive success and fetal health. In particular, lower MIF levels in early pregnancy have been associated with recurrent miscarriage, while higher levels in late pregnancy have been correlated with preeclampsia, the main cause of maternal and fetal mortality and morbidity worldwide [60, 61]. By altering the secretion of these two important molecules at the fetomaternal interface, BPA could contribute to the onset of pregnancy pathologies and therefore be detrimental for fetal and maternal health.

## 5. Conclusions 

By using* in vitro* models that mimic the maternal and fetal compartments in human pregnancy, we demonstrated that very low (0.5–1 nM) concentrations of BPA, an environmental chemical representing a hazard in the pre- and perinatal life, alter the placental secretion of hormone *β*-hCG and cytokine MIF, two key molecules of the immune-endocrine cross-talk at the fetomaternal interface. The endometrial stromal cells appear to play a protective role in the placenta, as no effects of BPA were detected when placental cultures were exposed to BPA-treated endometrial cells conditioned medium. Although the molecular mechanisms involved still remain unclear, the data obtained clearly revealed that the maternal endometrium is able to either accumulate or release BPA, depending on the surrounding environment. Considering that contamination with BPA occurs in everyday life, the potential effects that a long term endometrial accumulation of this chemical might have during pregnancy—on fetal/placental tissues—are a major concern.
